# Establishment of organoid models for pancreatic ductal adenocarcinoma and screening of individualized therapy strategy

**DOI:** 10.1002/ame2.12352

**Published:** 2023-10-27

**Authors:** Miaomiao Gong, Han Meng, Dengxu Tan, Peng Li, Jing Qin, Qingling An, Changhong Shi, Jiaze An

**Affiliations:** ^1^ Division of Cancer Biology, Laboratory Animal Center Fourth Military Medical University Xi'an China; ^2^ School of Basic Medical Sciences Medical College of Yan'an University Yanan China; ^3^ Animal Experiment Center Guangzhou University of Chinese Medicine Guangzhou China; ^4^ Department of Hepatobiliary and Pancreaticosplenic Surgery, Xijing Hospital Fourth Military Medical University Xi'an China

**Keywords:** 3D culture, individualized therapy, organoid, pancreatic ductal adenocarcinoma

## Abstract

**Background:**

Patients with pancreatic ductal adenocarcinoma (PDAC) who undergo surgical resection and receive effective chemotherapy have the best chance for long‐term survival. Unfortunately, because of the heterogeneity of pancreatic cancer, it is difficult to find a personalized treatment strategy for patients. Organoids are ideal preclinical models for personalized medicine. Therefore, we explore the cultivation conditions and construction methods of PDAC organoid models to screen the individualized therapy strategy.

**Methods:**

Fresh PDAC tissues from surgical resection were collected and digested with digestive enzymes; then the tumor cells were embedded in Matrigel with a suitable medium to establish the PDAC organoid models. The genetic stability of the organoids was analyzed using whole exon sequencing; hematoxylin and eosin staining and immunohistochemistry of organoids were performed to analyze their consistency with the pathological morphology of the patient's tumor tissue; After 2 days of organoid culture, we selected four commonly used clinical chemotherapy drugs for single or combined treatment to analyze drug sensitivity.

**Results:**

Two cases of PDAC organoid models were successfully established, and the results of their pathological characteristics and exome sequencing were consistent with those of the patient's tumor tissue. Both PDAC organoids showed more sensitivity to gemcitabine and cisplatin, and the combined treatment was more effective than monotherapy.

**Conclusion:**

Both organoids better retained the pathological characteristics, genomic stability, and heterogeneity with the original tumor. Individual PDAC organoids exhibited different sensitivities to the same drugs. Thus, this study provided ideal experimental models for screening individualized therapy strategy for patients with PDAC.

## Graphical abstract

We explored the cultivation conditions and construction methods of PDAC organoid models and successfully established two PDO models of PDAC. Both organoids better retained the pathological characteristics, genomic stability and heterogeneity with original tumor. Both organoids exhibited more sensitive to gemcitabine and cisplatin, and the combination treatment exhibited more effective than monotherapy. Particularly, Individual organoid exhibit different sensitivities to same drugs.

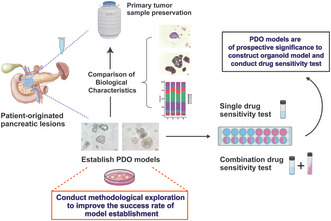

## 1 INTRODUCTION

In recent years, the incidence of pancreatic ductal adenocarcinoma (PDAC) has increased considerably.^1^ High malignancy and great variation in chemotherapy are the main reasons for the low 5‐year survival rate of pancreatic cancer, which is approximately 10%.^2^, ^3^ At present, individualized treatment for cancer patients can greatly improve survival time, so it is very important to establish the individualized drug screening model.^4^

In individualized drug treatment for pancreatic cancer, the main preclinical models are the patient‐derived xenograft (PDX) model and the patient‐derived organoid (PDO) model.^5^, ^6^ The former is time consuming, and it does not provide guidance to pancreatic cancer patients in a short time of disease progression. This model is not useful for guidance in personalized treatment.^7^, ^8^

Organoids are tissue‐ or organ‐like structures formed by the proliferation and differentiation of stem cells, which are 3D models in vitro culture.^9^ The PDO model requires the extraction of primary cells from patients' tumor tissue and the use of external growth factors as well as an exogenous extracellular matrix to create a 3D growth environment.^10^, ^11^, ^12^ This model is significantly advantageous over the traditional 2D cell models and the PDX model, and its phenotype and gene are similar to that of tumor epithelial cells.^12^ Organoids can simulate the pathological state of tumor cells in vitro and have a higher correlation with drug response in human clinical trials.^13^, ^14^, ^15^, ^16^ They provide a good platform for drug screening and detection of treatment plans for tumor patients. Furthermore, the discovery of gene mutations and drug therapy targets through deep sequencing or phenotype analysis is of great significance for the study of the pathogenesis and drug resistance of PDAC.

In this study, we focused on the cultivation conditions and construction methods of organoid models to explore the main factors that impact the success rate of organoid modeling. Two PDO models of PDAC were established, and their biological characteristics were confirmed to be consistent with the original tissue. We further selected four commonly used clinical chemotherapy drugs for single or combined treatment to analyze drug sensitivity in those two PDO models. The results showed that both PDAC organoids were more sensitive to gemcitabine (GEM) and cisplatin (CIS), and the combined treatment was more effective than monotherapy. Particularly, the individual PDO model exhibited different responses to the same drug.

## 2 MATERIALS AND METHODS

### 2.1 Specimen

The experimental procedures related to human pancreatic ductal carcinoma were performed under the supervision and guidance of the Ethics Committee of Xijing Hospital of the Fourth Military Medical University (FMMU). The ethics approval number was KY20203128‐1. The patient's informed consent was obtained for the acquisition of PDAC tissue. PDAC tissue was taken from a patient with PDAC after hepatobiliary surgery in Xijing Hospital of FMMU.

### 2.2 Configuration of medium for PDAC organoids

The organoid culture medium consisted of Advanced DMEM/F12, Primocin (100 μg/mL, Invitrogen)，HEPES (10 mM, Gibco), GlutaMAX‐I (1X, Gibco), A83‐01 (500 nM, Tocris), Y‐27632 (10 μM, AbMole), *N*‐acetylcysteine (1.56 mM, Sigma), nicotinamide (10 mM, Sigma), FGF10 (10 ng/mL, PeproTech), B27supplement (1X, Gibco), Epidermal Growth Factor (50 ng/mL, PeproTech), Gastrin I (0.01 μM, Tocris Bioscience), Wnt3A conditioned medium (50 ng/mL, R&D Systems)，R‐spondin‐1 conditioned medium (250 ng/mL, R&D Systems), and Noggin conditioned medium (100 ng/mL, R&D Systems).

### 2.3 Isolation and culture of PDAC organoids

The patient's tumor sample was removed from the necrotic area and ~0.5 cm^3^ of tumor tissue was rinsed twice with phosphate‐buffered saline. The specimen was cut using a human tumor separation kit (Miltenyi Biotec, Germany), and single cells were separated according to the instructions in Gentle MACS Octo Dissociator (Miltenyi Biotec); the whole process was performed in ~0.5–1 h. The tissue‐free solution was rinsed with phosphate‐buffered brine; the suspension was filtered through a cell filter (100 μm) and centrifuged at 300 *g* for 5 min. The supernatant was discarded, and the precipitated cells were collected and resuspended with an appropriate amount of matrix gel (356 231, Corning, USA); 50‐μL droplets were placed in six‐well plates and covered with an organoid medium. The cells were cultured in a humidified incubator with 5% CO_2_ at 37°C.

### 2.4 Whole exome sequencing

Total DNA was extracted using the DNeasy Blood and Tissue Kit (Qiagen) or QIAamp DNA FFPE Tissue (Qiagen). DNA sequencing library was constructed using Covaris M220–focused ultrasound (Covaris). Exome capture was performed using SureSelect Human All Exon V6 Kit (Agilent Technologies) following the vendor's recommended protocol. The sequencing was performed using an Illumina Novaseq 6000 system (LC‐Bio Technology Co., Ltd.) in 150‐bp paired‐end sequencing mode.

### 2.5 Histopathology staining

Tumor tissues and organoids were fixed in 4% paraformaldehyde (Servicebio, China) for 24 h. Paraffin‐embedded specimens were cut into 5‐μm sections for hematoxylin and eosin, immunohistology, and immunofluorescence staining. Immunohistohistological staining was performed with different antibodies, including HER2 specific antibody (2165S, Cell signaling, 1:400), CA19‐9 specific antibody (ab398, Abcam, 1:100), CEA specific antibody (ab133633, Abcam, 1:300), EGFR specific antibody (ab32198, Abcam, 1:100), and Ki67 specific antibody (ab16667, Abcam, 1:200).

### 2.6 Drug screening

Organoid cells were inoculated at 5 × 10^3^ cells/well, and the medium was changed after 1–2 days of culture. Four commonly used chemotherapy drugs in clinical practice, namely GEM, CIS, irinotecan (CPT‐11), and 5‐fluorouracil (5‐FU), were selected for single treatment and combined treatment (GEM combined with CIS or GEM combined with 5‐FU). The drug was dissolved according to instructions. Each drug was diluted in an organoid medium with different dilution ratios (Table S1), and the maximum dose was capped by the maximum plasma concentration reported by patients.^17^ The single‐drug concentration settings in the combination therapy are the same as those in the single‐drug therapy. Cell viability was measured using the Cell Titer‐Glo 3D kit (Promega, USA).

### 2.7 Statistical analysis

GraphPad Prism 9 was used for statistical analysis. All data were performed using two‐tailed unpaired *t*‐tests. Differences between three or more conditions with one independent variable were analyzed using one‐way analysis of variance; *p*‐values were set as * < 0.05 and ** < 0.01. Image J software was used for analyzing the results of immunofluorescence and determining the mean gray value.

## 3 RESULTS

### 3.1 Establishment of PDAC organoids

**FIGURE 1:**
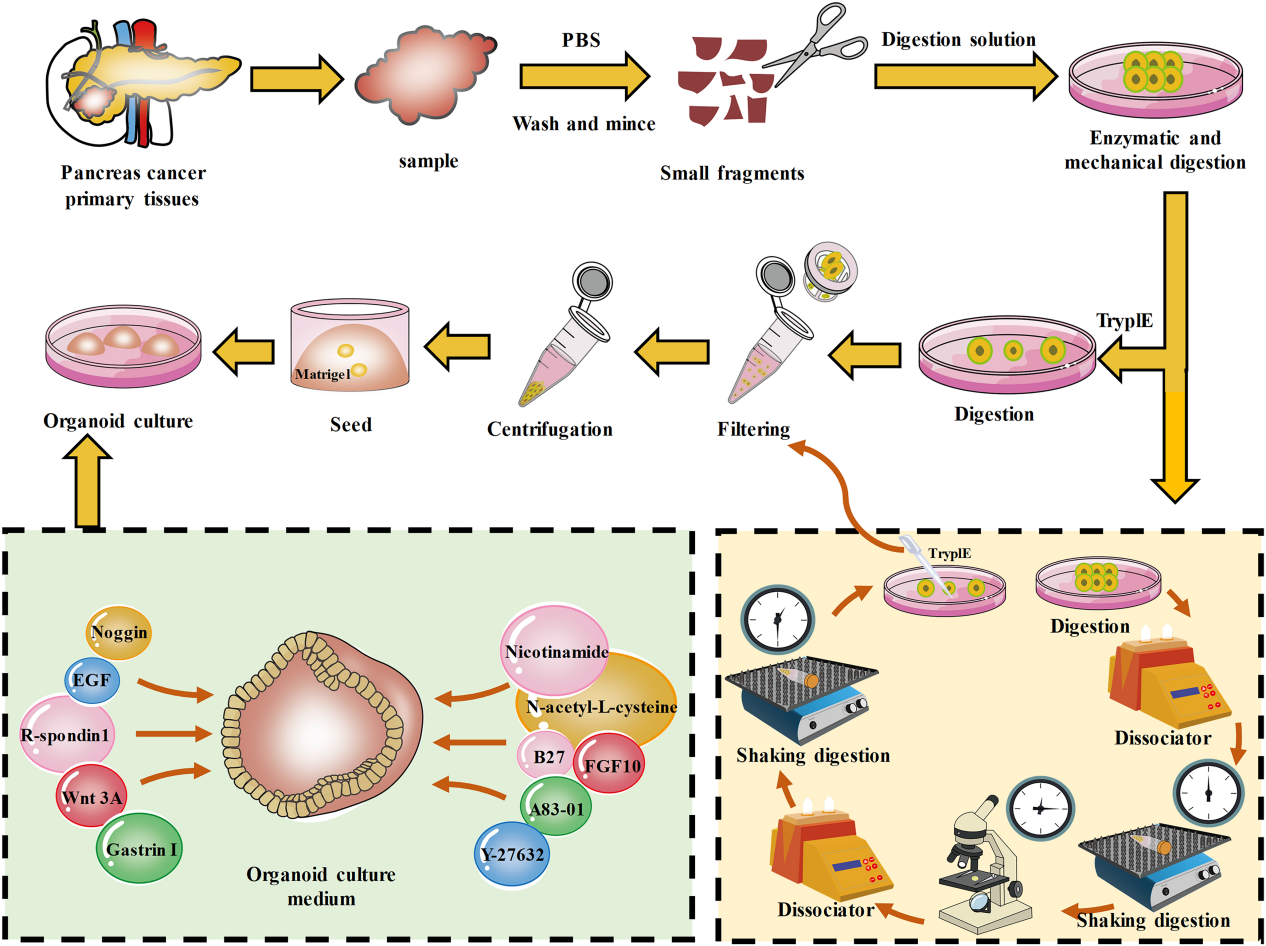
Flowchart for establishment of pancreatic cancer organoid model. The main operation process of establishment of pancreatic cancer organoid model; schematic representation of the main components of the pancreatic cancer organoid culture medium; detailed steps during digestion of pancreatic cancer organoids.

Two organoid models of PDAC from three clinical specimens were successfully established based on the main operating flowchart (Figure 1). All patients were undergoing a standard pancreatectomy. Postoperative pathological analysis showed both cases were PDAC with moderate and high differentiation (Table S2).

In the process of establishing the model, several details may directly affect the success rate of the model. First, the tumor sample should be obtained as soon as possible to ensure the activity of the tumor cells. When a tissue dissector was used for grinding, different procedures were set based on the softness and hardness of the specimen. Usually, the digestion time of pancreatic cancer tissue ranged from 0.5 to 1.0 h. For harder or tougher tumor tissues, the digestion time should be appropriately prolonged. The most important factor is to ensure the viability of the tumor cells and try to digest the tumor cells into single cells. For softer tumor tissues, the digestion time can be reduced to fully ensure the viability of the tumor cells. The digestion process needs to be dynamically observed under the microscope, and it must be terminated at the appropriate time.

After the tumor cells are obtained, the additional amount of Matrigel should be determined based on the amount of cell precipitates, and the cell count usually was controlled within 3 × 10^3^ to 5 × 10^3^ cells/well. The dome‐like gel drops formed by cell suspension should be controlled within 40–50 μL. Very large droplets are not conducive for absorption of the medium and may cause the gel droplet to rupture. If the droplet is too small, it will cause a waste of medium. After inoculation in the culture plate, it should be quickly inverted and placed in a 37°C incubator for culture. The control time was commonly ~15 min. A very short time will cause the gel droplets to not coagulate effectively and result in the destruction of the three‐dimensional structure; a very long time will cause the cells to disassemble and result in cell death due to the lack of sufficient medium.

### 3.2 Pathological features of PDAC organoids

**FIGURE 2:**
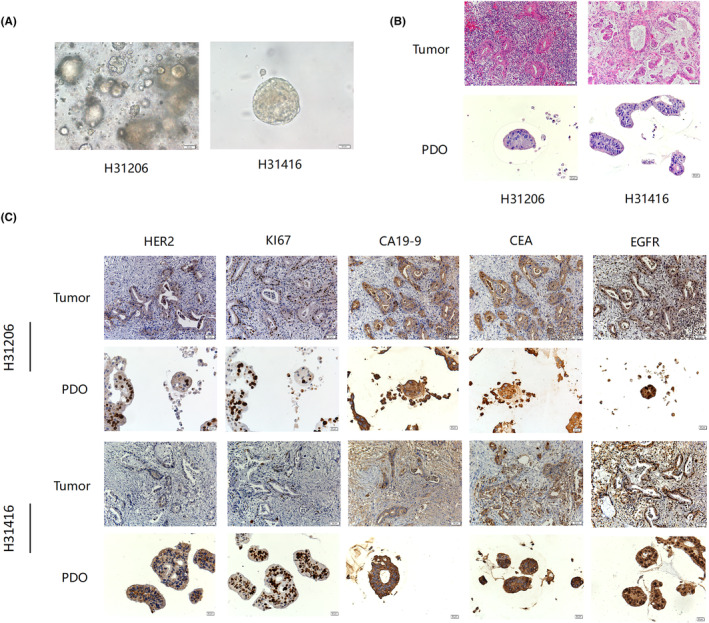
Pathological features of PDAC (pancreatic ductal adenocarcinoma) organoid model. (A) Representative images of two pancreatic tumor organoids (scale bar, 50 μm). (B) H&E staining of the patient tumor (scale bar, 50 μm), PDO (patient‐derived organoid) samples (scale bar, 20 μm). (C) The expression of tumor markers was detected using immunohistochemistry in patient tumor (scale bar, 50 μm) and PDO samples (scale bar, 20 μm). H&E, hematoxylin and eosin staining.

We obtained images every other day to record the growth status of the organoid using an optical microscope. On the first day after inoculation, PDAC cells clustered, or even individual cells grew and divided to assemble into cell clusters. At this time, they were solid and then continued to grow and differentiated into a cavity (Figure 2A). The heterotypic characteristics of the organoid nuclei in PDAC were highly consistent with the hematoxylin and eosin staining of parental tissues (Figure 2B). Further analysis of its pathological features revealed positive results for tumor‐related biomarkers, such as CA19‐9, CEA, EGFR, Ki67, and HER2 (Figure 2C). These results indicated that the established organoids derived from patients lacked extracellular‐related components in tissue morphology, the organoid components were relatively single, and the majority were epithelial cells of PDAC. However, in terms of pathological features, organoids effectively preserved various tumor‐related biomarkers in the tumor tissue of patients.

### 3.3 PDAC organoid model retains the genetic characteristics of pancreatic tumor tissues

**FIGURE 3:**
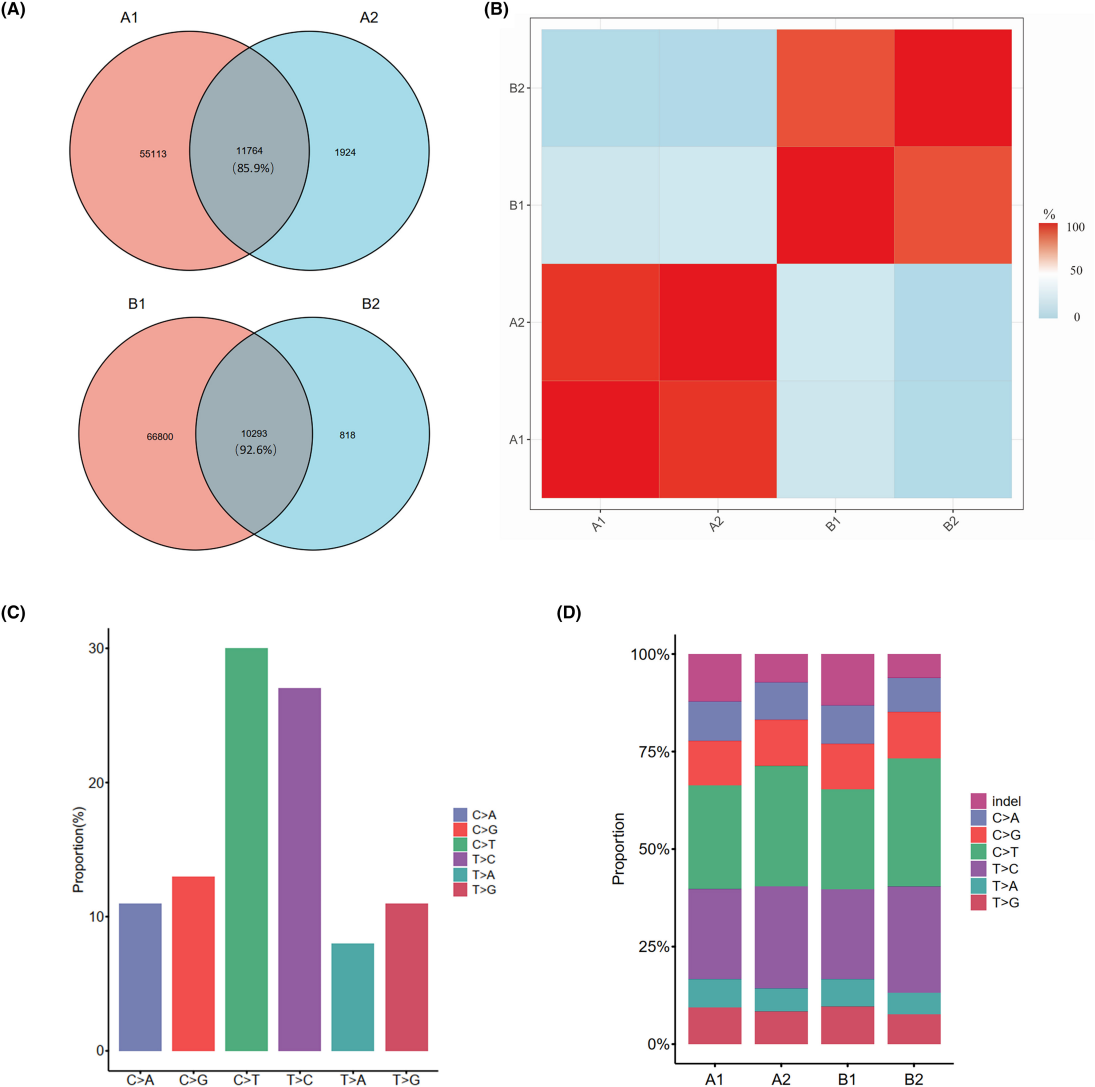
PDAC (pancreatic ductal adenocarcinoma) organoid model retains the main genetic characteristics of pancreatic tumor tissues. (A and B) Correlation heat map between the variants identified in patient tumor and corresponding organoids. Patient‐derived tumor tissue was analyzed in these two organoids, with mutated genes accounting for 85.9% and 92.6% of the organoid genes, respectively. (C) Overall distribution of base substitutions detected in all samples, including organoids and the corresponding tissues. (D) Proportion of exonic variations detected in each organoid and the corresponding tissues. A1: the tumor tissue of H31206 patient; B1: the tumor tissue of H31416 patient; A2: H31206 organoid; and B2: H31416 organoid.

To determine whether organoids retained the mutation characteristics of patient tissues, whole exome sequencing analysis was performed on the organoid cultures and the corresponding primary tissues. A comparison of genomic changes showed that the mutation profiles in these two organoids and patient‐derived tumor tissue were better retained, with mutated genes accounting for 85.9% (H31206) and 92.6% (H31416) of the organoid genes, respectively (Figure 3A,B), indicating that the established PDAC organoid preserved the genetic characteristics of patient‐derived tumor tissue. The distribution of base substitutions in exons of tissues and organoids was analyzed, and the results showed that C > T accounted for the highest proportion, followed by T > C and C > G, whereas C > A, T > G, and T > A accounted for relatively less (Figure 3C). Two organoids were compared separately with tumor tissues from corresponding patient specimen. The proportion of base mutations in various parts of the organoids cultured in vitro was consistent with that of the patient tissue. When analyzed separately, the proportion of C > T in organoids was higher than that of the patient tissue. We believed this was related to the environment of organoids cultured in vitro (Figure 3D).

### 3.4 Sensitivity to a single drug in PDAC organoids

**FIGURE 4:**
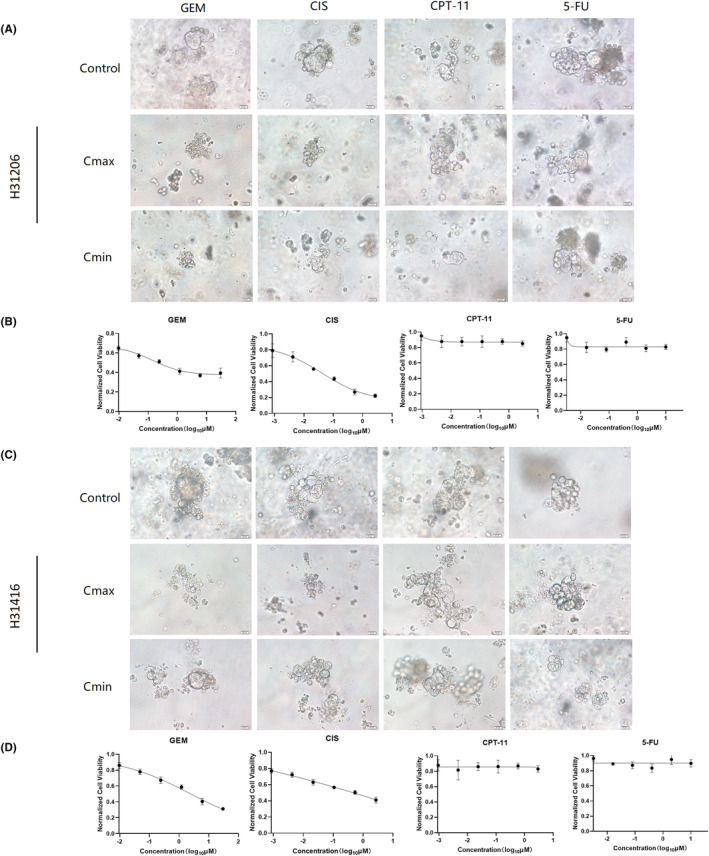
Single‐drug screening in PDAC (pancreatic ductal adenocarcinoma) organoid model. (A) Bright‐field microscopy images of H31206 PDO (patient derived organoid) model treated with four drugs (GEM [gemcitabine], CIS [cisplatin], 5‐Fu [5‐fluorouracil], and CPT‐11 [irinotecan]), including controls, *C*_max_, and *C*_min_ (scale bar, 20 μm). (B) Cell viability of H31206 PDO treated with different concentrations of drugs. (C) Bright‐field microscopy images of H31416 PDO model treated with four drugs (GEM, CIS, 5‐Fu, and CPT‐11), including controls, *C*_max_, and *C*_min_ (scale bar, 20 μm). (D) Cell viability of H31416 PDO treated with different concentrations of drugs. *C*_max_, maximum concentration; *C*_min_, minimum concentration.

The organoid model is a new tool for predicting drug efficacy and drug discovery and is suitable for high‐throughput drug selection. Tumor organoid had become a preclinical cancer model for drug discovery and screening, with great potential for predicting the therapeutic effects of chemotherapy drugs. To explore the potential of organoid screening for personalized treatment, we selected four commonly used clinical chemotherapeutic drugs, GEM, CIS, CPT‐11, and 5‐FU, to test drug sensitivity. Those two PDAC organoids were inoculated in 96‐well plates and cultured for 2 days, and four drugs were added. It was observed that both cases were more sensitive to GEM and CIS (Figure 4A,C). Unlike 5‐FU and CPT‐11 drugs, after the action of GEM and CIS, the cells appeared to disintegrate to various degrees with the increase in drug concentration; however, when treated with 5‐FU and CPT‐11, the cell activity did not change significantly with the increase in drug concentration, and the cell morphological integrity was higher. The Cell Titer Glo 3D kit was further used to detect organoid activity, which was consistent with the microscopic observation results; both models were sensitive to GEM and CIS (Figure 4B,D); H31206 organoid was the most sensitive to GEM, whereas H31416 organoid was the most sensitive to CIS (Figure 4B,D).

### 3.5 Sensitivity to combination therapy in PDAC organoids

**FIGURE 5:**
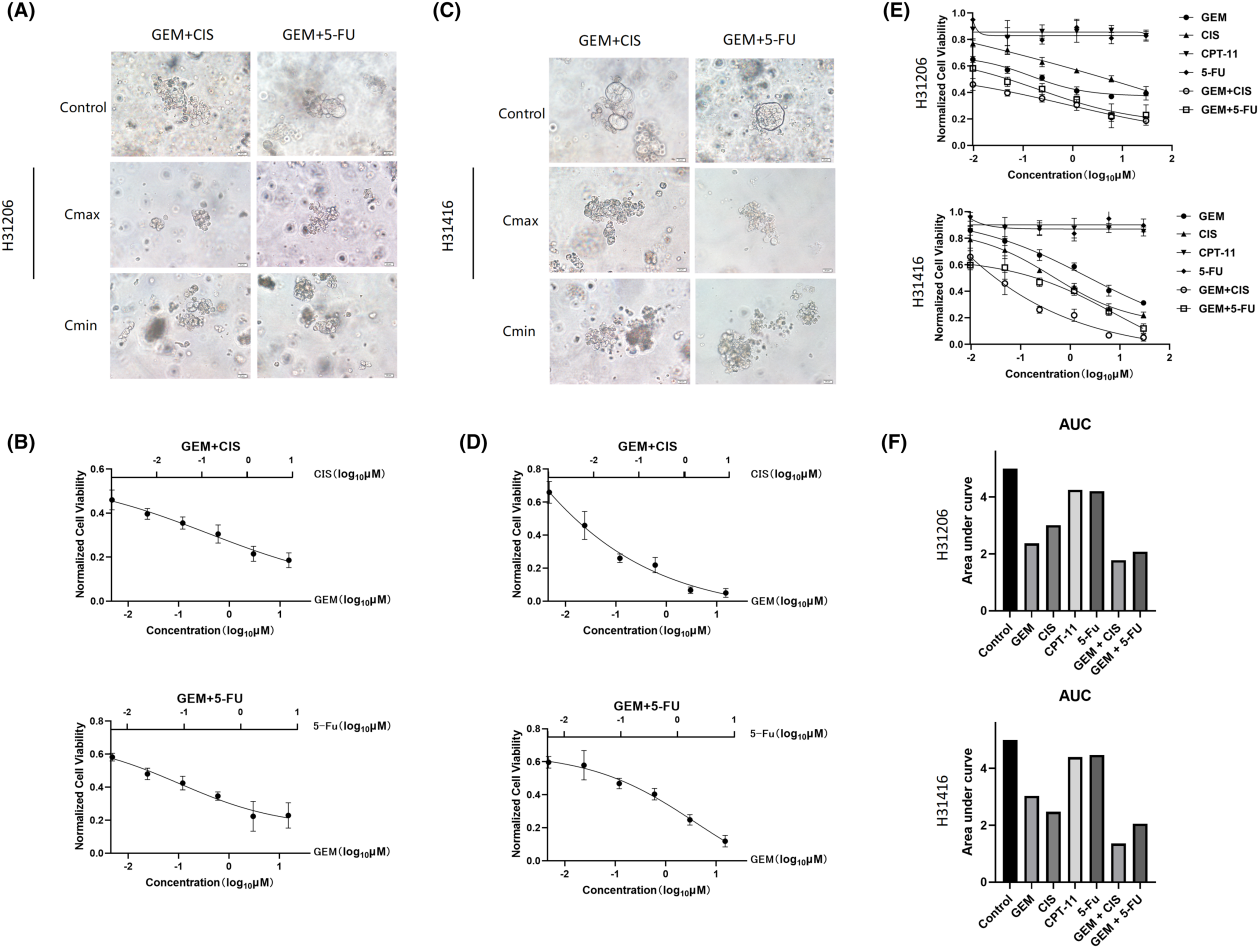
Combination treatment strategies screening in PDAC (pancreatic ductal adenocarcinoma) organoid model. (A) Bright‐field microscopy images of H31206 PDO (patient derived organoid) model treated with two groups of combination drugs (GEM + CIS, GEM +5‐Fu) including controls, *C*_max_, and *C*_min_ (scale bar, 20 μm). (B) Cell viability of H31206 PDO treated with different concentrations of combination drugs. (C) Bright‐field microscopy images of H31416 PDO model treated with two groups of combination drugs (GEM + CIS, GEM +5‐Fu), including controls, *C*_max_, and *C*_min_ (scale bar, 20 μm). (D) Cell viability of H31416 PDO treated with different concentrations of combination drugs. (E) PDO cell viability with single and combination drugs. (F) Area under the curve (AUC) of PDO cell viability for single and combination drugs. CIS, cisplatin; *C*_max_, maximum concentration; *C*_min_, minimum concentration; GEM, gemcitabine.

In clinical treatment of PDAC, patients with acquired drug resistance are generally treated with multidrug combination therapy. In the single‐drug sensitivity test, we found that GEM and CIS were more sensitive. Therefore, we selected a combination of GEM and CIS for treatment; in addition, GEM was the preferred drug for the treatment of PDAC; thus, a combination therapy with 5‐FU, which is not sensitive to single‐drug therapy, was also chosen. The results showed that the combination therapy of GEM and CIS was more effective. We observed that both cases were more sensitive to the combination therapy of GEM and CIS than monotherapy, and the cells exhibited varying degrees of disintegration as the drug concentration increased; in the combination therapy of GEM and 5‐FU, cell activity also changed with the increase in drug concentration, but the degree of cell disintegration was lower than that of the combination therapy of GEM and CIS (Figure 5A,C). Subsequently, organoid activity was detected using the Cell Titer Glo 3D kit, and the results demonstrated that both cases were sensitive to the combination therapy of GEM and CIS, which was consistent with the results of microscopic observation (Figure 5B,D). In addition, we evaluated the area under the curve (AUC) of various drugs for quantifying drug reactions in organoids. The AUC represents the sensitivity of PDO to different drugs.^17^ Consistent with previous results, two cases of organoids showed different reactions to the same drugs, which also reflected the heterogeneity of the organoid models in different patients (Figure 5E,F).

## 4 DISCUSSION

The main reason for the low survival rate of patients with PDAC is the heterogeneity of tumor, which leads to the inconsistent response of chemotherapeutic drugs.^18^, ^19^ Therefore, it is particularly important to select an accurate drug treatment strategy. The PDO model is an ex vivo three‐dimensional model derived directly from patients' tumor tissues. It maintains the pathohistological and genetic characteristics of patient tumors and preserves tumor heterogeneity. Vlachogiannis et al.^4^ reported that compared to clinical patient reactions the sensitivity of the gastrointestinal organoids to drugs was as high as 100%, the specificity was 92%, and the positive prediction rate was 88%. Thus, this model exhibits unique advantages in drug screening and prediction of clinical patient response. In addition to PDO modeling, PDX is widely used in clinical patient drug screening.^7^, ^20^, ^21^ Compared with the PDX model, the PDO model can be established at a lower cost, and the time from the establishment of the PDO model to the completion of the drug trial is short; also it is easy to be cryopreserved, which is a challenge for the PDX model. Therefore, the PDO model is more suitable for individualized treatment of tumor.^22^, ^23^

In this study, we explored the main aspects that impact the establishment of organoid modeling, including activity of cells, digestion time, amount of Matrigel, cell count, dome‐like gel drops, and inoculation time, which are significant guiding factors for other subsequent personnel engaged in PDO modeling. Based on these aspects, we successfully established two PDAC organoid models with the ability to proliferate in vitro. Those two organoids exhibit different sensitivities to the same drugs.

The sequencing results demonstrated that the genetic stability of the primary tumor was preserved in both cases. The PDAC‐related markers CA19‐9, CEA, EGFR, Ki67, and HER2 in the organoid tissues were consistent with those of patient tumor tissues, suggesting that these two PDO models better maintained the clinical characteristics of primary tumors. In the process of PDAC organoid culture, the shape of the PDAC organoid was basically the same; most of them were cystic. Even though the size of the cell mass was different at inoculation, with continuous cell growth, the single cell and small cell mass gradually turned into cavities, and the larger cell mass also formed cavities like organoids. These results showed that most of the organoids of PDAC will eventually grow into a cavity, which is consistent with the characteristics of most PDAC organoid morphology.^24^, ^25^, ^26^

GEM, CIS, CPT‐11, and 5‐FU are commonly used in clinical chemotherapy for PDAC. Of these, GEM is the first choice for clinical treatment of PDAC. The two PDO models in this study are sensitive to both GEM and CIS, but there were certain differences in different patients, which further reflects the heterogeneity and individualization of the PDO models. The combination strategy based on GEM showed a better therapeutic effect, which was consistent with clinical drugs, reflecting the clinical similarity of the PDO model for drug screening. The confirmation of drug concentration is crucial for drug screening in the PDO model. We usually select the maximum plasma concentration reported by patients as the maximum dose^18^ or refer to the relative references to obtain the concentration to further determine the IC_50_ values based on the reaction of PDO.

How to improve the success rate of models is the key to PDO research. It was reported that optimizing the culture conditions and incorporating other advanced cell/tissue culture technologies could increase the success rate of PDO establishment.^7^, ^9^ In this study, we also found that malignancy and freshness of sample were the two main factors affecting the model, because more malignant tumors always proliferate faster and more fresh tumor cells exhibit better activity.^27^ Meanwhile, the process of tumor digestion is also critical. The digestion time of different quality specimens could directly determine cell activity. We should dynamically adjust the digestion time based on the quality of the specimen. Comprehensively considering these influencing factors, we successfully established two PDO models from three clinical specimens, and the success rate reached about 66%, almost consistent with literature reports.^15^, ^28^, ^29^

Although the organoid model has well preserved the integrity of tumor heterogeneity, it also has certain limitations, such as the lack of internal environment, including blood vessels, blood, immunity, and metabolism. Currently, it has been reported that coculture with immune cells or fibroblasts and microfluidic chips can compensate this disadvantage.^30^, ^31^, ^32^, ^33^, ^34^ The coculture system of organoid cells with immune cells can also be established based on James's relevant research for screening immune checkpoint inhibitors, and the coculture model of PDO with peripheral blood mononuclear cells can also be applied to the evaluation of immunotherapy drugs.^32^ Targeted refinement of the PDO modeling approach is important not only for the modeling success, but also for further refinement of organoid functionality.

In summary, two cases of PDAC organoid models were successfully established. Both organoids better retained the pathological characteristics, genomic stability, and heterogeneity with the original tumor. Individual PDAC organoids exhibited different sensitivities to the same drugs. Thus, this study provided ideal an experiment model for screening the individualized therapy strategy for PDAC patients. Further, we should establish more PDO models to verify our findings; also all drug screening results of PDO models should be validated clinically.

## AUTHOR CONTRIBUTIONS

Miaomiao Gong: conceptualization, methodology, data curation, and writing—original draft; Han Meng: investigation, data curation, and formal analysis; Dengxu Tan: investigation, data curation, and formal analysis; Peng Li: investigation and visualization; Qingling An and Jing Qin: visualization and writing—review and editing; Jiaze An: investigation. Changhong Shi: conceptualization, writing, supervision, and funding acquisition. All authors have read and agreed to the published version of the manuscript.

## CONFLICT OF INTEREST STATEMENT

The authors declare that they have no known competing financial interest or personal relationships that could have appeared to influence the work reported in this paper.

## ETHICAL APPROVAL

This study was approved by the Ethics Committee of Xijing Hospital of FMMU (ethics approval number: KY20203128‐1).

## Supporting information


Tables S1–S2

## Data Availability

The data that support the findings of this study are available from the corresponding author on reasonable request.
