# Supplementary material for: Establishment of organoid models for pancreatic ductal adenocarcinoma and screening of individualized therapy strategy
Source: Animal Model Exp Med. 2023 Oct 27;6(5):409–18. doi: 10.1002/ame2.12352 (PMC10614126; doi:10.1002/ame2.12352)
Supplement: Supplementary file 1 — Tables S1–S2 [file AME2-6-409-s001.docx]

# Supplementary Material

**Table 1.**  Specific concentration of drugs acting on organoids

| Group | Drug Concentrations | | | | | |
| --- | --- | --- | --- | --- | --- | --- |
| GEM | 30μM | 6μM | 1.2μM | 240nM | 48nM | 9.6nM |
| CIS | 2.67μM | 534nM | 106.8nM | 21.36nM | 4.27nM | 0.85nM |
| 5-Fu | 10μM | 2μM | 400 nM | 80 nM | 16nM | 3.2nM |
| CPT-11 | 2.95uM | 590nM | 118nM | 23.6nM | 4.72nM | 0.94nM |

**Table S2.** General information of patients with pancreatic ductal adenocarcinoma

| Number | Gender | Age | Categories of Tumors | Tumor location | pathological stage | Grade of clinical |
| --- | --- | --- | --- | --- | --- | --- |
| H31206 | female | 64 | PDAC | pancreas | AJCC pT2N1 | G2 |
| H31416 | male | 60 | PDAC | pancreas | AJCC pT2N0 | G2-G3 |
